# Regulation of endogenous neural stem/progenitor cells for neural repair—factors that promote neurogenesis and gliogenesis in the normal and damaged brain

**DOI:** 10.3389/fncel.2012.00070

**Published:** 2013-01-18

**Authors:** Kimberly J. Christie, Ann M. Turnley

**Affiliations:** Neural Regeneration Laboratory, Department of Anatomy and Neuroscience, Centre for Neuroscience Research, The University of MelbourneParkville, VIC, Australia

**Keywords:** neural stem cell (NSC), neural precursor cell (NPC), neural stem/precursor cell (NSPC), neurogenesis, gliogenesis, traumatic brain injury, ischemic stroke, subventricular zone (SVZ)

## Abstract

Neural stem/precursor cells in the adult brain reside in the subventricular zone (SVZ) of the lateral ventricles and the subgranular zone (SGZ) of the dentate gyrus in the hippocampus. These cells primarily generate neuroblasts that normally migrate to the olfactory bulb (OB) and the dentate granule cell layer respectively. Following brain damage, such as traumatic brain injury, ischemic stroke or in degenerative disease models, neural precursor cells from the SVZ in particular, can migrate from their normal route along the rostral migratory stream (RMS) to the site of neural damage. This neural precursor cell response to neural damage is mediated by release of endogenous factors, including cytokines and chemokines produced by the inflammatory response at the injury site, and by the production of growth and neurotrophic factors. Endogenous hippocampal neurogenesis is frequently also directly or indirectly affected by neural damage. Administration of a variety of factors that regulate different aspects of neural stem/precursor biology often leads to improved functional motor and/or behavioral outcomes. Such factors can target neural stem/precursor proliferation, survival, migration and differentiation into appropriate neuronal or glial lineages. Newborn cells also need to subsequently survive and functionally integrate into extant neural circuitry, which may be the major bottleneck to the current therapeutic potential of neural stem/precursor cells. This review will cover the effects of a range of intrinsic and extrinsic factors that regulate neural stem/precursor cell functions. In particular it focuses on factors that may be harnessed to enhance the endogenous neural stem/precursor cell response to neural damage, highlighting those that have already shown evidence of preclinical effectiveness and discussing others that warrant further preclinical investigation.

## Introduction

The concept that the adult mammalian brain contains populations of resident neural stem/progenitor cells (hereafter collectively referred to as NSPCs) was generally accepted two decades ago (Reynolds and Weiss, 1992; Richards et al., 1992), although first shown by Altman and Das in 1965 (Altman and Das, 1965). Adult neurogenesis occurs primarily in two neurogenic regions in the brain, the subventricular zone (SVZ) of the lateral ventricles and the subgranular zone (SGZ) of the dentate gyrus in the hippocampus. To produce new neurons a NSPC must go through a process of proliferation, migration, differentiation, and integration to become a productive member of existing circuitry in the brain. Under normal physiological conditions adult NSPCs predominantly produce neurons, in particular interneurons in the olfactory bulb (OB) for SVZ-derived cells and dentate gyrus granule cell neurons for SGZ-derived cells. Following neural damage, however, SVZ-derived cells have the capacity to respond to the insult and produce neurons as well as astrocytes and oligodendrocytes. Harnessing this ability of NSPCs to produce new neuronal and glial cells as a means to replace cells damaged or lost following neural injury or disease to promote neural repair has been the focus of a substantial body of research. There are four main areas in the life cycle of a NSPC that can be targeted to try to enhance neural repair, namely proliferation, migration, differentiation (and specific cell type generation), and survival/integration. However, the field is full of conflicting reports on the benefit and ability of NSPCs to recover function following a range of injuries and diseases. While many labs have reported an increase in proliferation of neuroblasts and even migration to injured areas, a large part of the problem may lie in the failure of newly generated neurons to integrate into existing neural circuitry and survive past 4 weeks or so after their generation. Augmentation of all steps of the process of neurogenesis would ideally lead to an increased benefit following injury, but ultimately the cells must integrate and survive to have a functional benefit.

**Figure 1:**
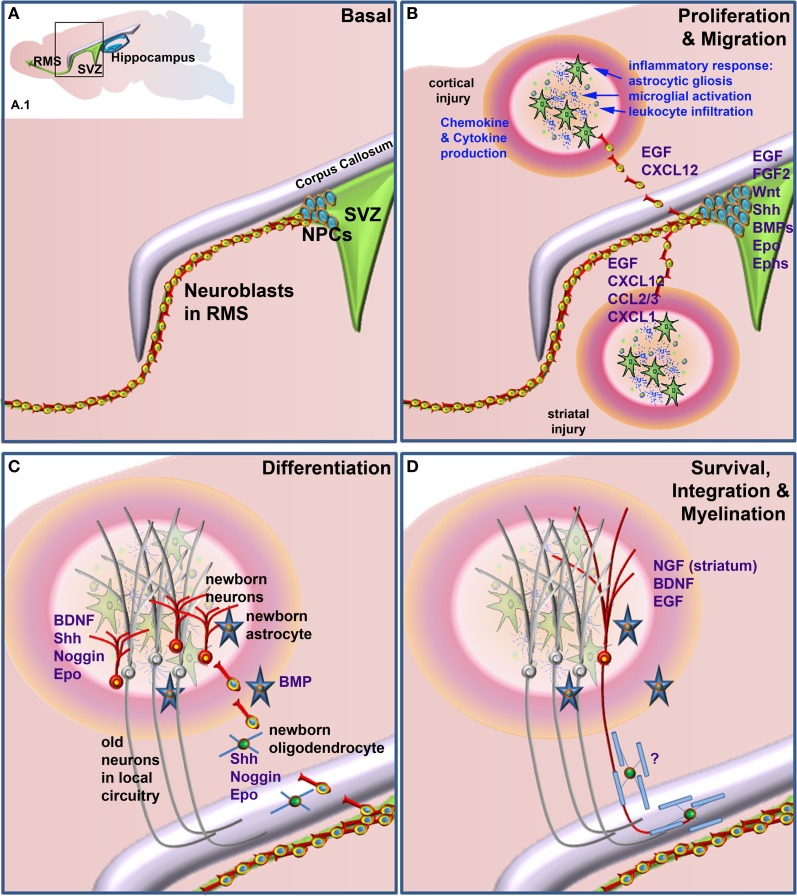
**Factors that when administered to the brain following neural damage regulate neural precursor cell responses. (A)** Basal, non-damage conditions. Inset **(A.1)** depicts the two main neurogenic regions of the adult rodent brain, the subventricular zone (SVZ) and the hippocampus. The enlarged area bounded by the box in **(A.1)** depicts the general structure of the SVZ, which contains proliferative neural precursor cells (NPCs) that differentiate into neuroblasts that then migrate along the rostral migratory stream (RMS) to the olfactory bulb. Following neural injury **(B)** the inflammatory response induces astrocytic gliosis, microglial activation, and leukocyte infiltration from the periphery. Together, these produce a variety of chemokines, cytokines and other factors that can lead to the proliferation of the NPCs in the SVZ and redirect their migration to sites of damage. Once at or near the site of damage **(C)** these cells can differentiate into astrocytes, oligodendrocytes and astrocytes, depending upon which factors are administered. Unlike the normal predominance of neuronal differentiation under basal conditions, following injury SVZ NSPCs can generate neurons, astrocytes and/or oligodendrocytes. **(D)** Following differentiation cells need to survive and appropriately integrate into existing circuitry. This is a large bottleneck in harnessing the therapeutic potential of NSPCs, as the majority of newborn neurons die within a few weeks of their generation. For correct function, many new neurons also need to be appropriately myelinated although at present, few if any factors are known that enhance the final myelination stage of oligodendrocyte maturation following neural damage. Factors that have been shown to regulate the various stages of the NPC response when administered following neural injury are listed in blue text.

This review will cover some of the main factors known to be involved in neurogenesis and gliogenesis in the adult CNS and in particular those that have been shown to play a role in neural repair. It will also explore how inflammatory mediators and other injury-related factors may modify the NSPC response to neural damage. We will not undertake a comprehensive review of factors regulating neurogenesis and gliogenesis in the normal developing or adult brain, as there have been several recent and comprehensive reviews covering this area (e.g., Guillemot, 2007; Ming and Song, 2011; Faigle and Song, 2012). Rather, we will highlight factors that play a role in regulation of normal adult NSPC function and which have been shown to be modulated to enhance repair following neural damage. Table 1 summarizes the factors discussed, which NSPC populations are affected (SVZ or SGZ), the type of neural injury, if any and the NSPC function affected. Figure 1 depicts the effects of extrinsic factors on the SVZ-derived NPC response to injury.

**Table 1:** **Extrinsic factors affecting SVZ and SGZ NSPC responses under basal conditions and following neural damage**.

**Factor**	**SVZ**				**SGZ**				**Injury/Disease**	**Neurogenesis/Gliogenesis**	**Selected references**
	**P**	**M**	**D**	**S**	**P**	**M**	**D**	**S**			
EGF	✓	✓			✓			X	Stroke, TBI	Both-variable	(Teramoto et al., 2003; Sun et al., 2010)
EGF+/−FGF2	✓				✓			✓	Ischemia, TBI, PD	N	(Nakatomi et al., 2002; Tureyen et al., 2005; Winner et al., 2008; Sun et al., 2009)
ATP	✓										^*^(Suyama et al., 2012)
VEGF	✓	✓									^*^(Wittko et al., 2009; Calvo et al., 2011)
IGF1		✓			✓			✓		N (SGZ)	^*^(Aberg et al., 2000; Cheng et al., 2001)
NGF								✓	Ischemia	N	(Zhu et al., 2011)
BDNF			✓	✓			✓	✓	Ischemia, TBI	N	(Gao and Chen, 2009; Im et al., 2010)
Wnt (β-catenin)	✓		✓?	✓?	✓				Ischemia, TBI, AD	both	(He and Shen, 2009; White et al., 2010; Shruster et al., 2012)
Shh	✓				✓				Ischemia, SCI	both	(Bambakidis et al., 2003, 2012; Sims et al., 2009; Wang et al., 2009)
BMPs (noggin)	✓		✓		✓		✓		Demyelination TBI, intra-ventricular hemorrhage	BMP: A, Noggin: N and O	(Hampton et al., 2007; Cate et al., 2010; Dummula et al., 2011; Sabo et al., 2011; Lei et al., 2012)
Epo	✓	✓	✓		✓			X	Stroke, TBI, PD	N and O	(Tsai et al., 2006; Wang et al., 2006; Kadota et al., 2009; Meng et al., 2011; Ning et al., 2011; Xiong et al., 2011; Zhang et al., 2012)
SOCS2			✓					✓		N	^*^(Turnley et al., 2002; Ransome and Turnley, 2008b)
Chemokines	✓	✓		✓	✓						(Imitola et al., 2004; Gordon et al., 2009)
Eph/ephrins	✓	✓	✓						TBI, ischemia, PD	N	(Theus et al., 2010; Doeppner et al., 2011; Jing et al., 2012)
Nitric oxide	✓				✓				Ischemia		^*^(Packer et al., 2003; Park et al., 2003; Moreno-Lopez et al., 2004) (Zhang et al., 2001a; Zhu et al., 2003; Sun et al., 2005b)
Endo-cannabinoids	✓	✓			✓	✓				N	^*^(Aguado et al., 2005, 2007; Jiang et al., 2005; Goncalves et al., 2008; Gao et al., 2010; Hill et al., 2010; Wolf et al., 2010; Oudin et al., 2011a,b)
PSA-NCAM, Slit-Robo, integrins		✓									^*^(Cremer et al., 1994; Hu et al., 1996; Jacques et al., 1998; Wu et al., 1999; Chazal et al., 2000; Murase and Horwitz, 2002)
Reelin		✓				✓			Demyelination	N-migration	(Courtes et al., 2011)
Vasculature		✓							Ischemia	N-migration	(Ohab et al., 2006; Yamashita et al., 2006; Thored et al., 2007; Kojima et al., 2010)
NMDA receptors				✓		✓		✓		N	^*^(Platel et al., 2010; Namba et al., 2011)

P, Proliferation; M, Migration; D, Differentiation; S, Survival; N, Neurogenesis; O, Oligodendrogliogenesis; A, Astrogliogenesis; TBI, traumatic brain injury; PD, Parkinson's disease; AD, Alzheimer's disease; SCI, spinal cord injury;

* references for basal non-injury conditions.

### NSPC fate: normal neurogenic niches vs. the injured CNS environment

Although the bulk of neurogenesis and gliogenesis occurs during embryonic and early postnatal development, NSPCs continue to produce neural cells in the adult brain. Importantly for the purpose of harnessing adult NSPCs for neural repair, unlike during development, the vast majority of adult-derived cells are fated to a neuronal lineage, with a much smaller percent differentiating into oligodendrocytes in the normal adult brain. In general, the diversity of cell types and neuronal subtypes that can be spontaneously generated by adult NSPCs is substantially limited compared to embryonic cells. This is probably largely due to a less permissive adult environment rather than a constitutive feature of the NSPCs themselves, as under appropriate conditions, such as in neurospheres *in vitro* (Reynolds and Weiss, 1992) or following ablation of neuronal populations without inflammatory system activation (Magavi et al., 2000), the ability of adult NSPCs to produce different neural cell types has been demonstrated. The more restricted fate of adult NSPCs in the normal adult CNS reflects the relative paucity of growth and neurotrophic factors compared to during development. The presence of inhibitory/attractive substrates, such as in the RMS, to constrain migratory routes and the lack of available space in the adult circuitry to allow integration of newborn cells, in general restricts normal NSPC function to neurogenic regions and currently limits the ability of endogenous NSPCs to replace specific neuronal or glial types in different regions in the CNS.

To further complicate matters, the injured adult CNS is an entirely different environment to the normal adult neurogenic niche or the developing nervous system, with substantial influence on NSPC function that in some instances appear to override the normal program of NSPC fate. This is particularly the case for SVZ NSPCs, which, as detailed further below, can be induced away from their normal migratory route to the OB toward the site of neural damage, a process largely induced by inflammatory mediators. After successful migration to the correct location, new neuroblasts must differentiate into the proper phenotype of neuron and integrate into local circuitry. The local circuitry to be repaired will depend on the type of damage, be it ischemic, traumatically injured or neurodegenerative, with some common factors and others specific to the site and type of damage. Effects of exogenous factors have been variably examined in each of the above types of neural damage and are described below for the relevant factors.

The majority of research on ectopic migration and neural differentiation of SVZ-derived NSPCs following neural damage has been performed by use of ischemia models and has demonstrated that cells do indeed reach the injured parenchyma (Arvidsson et al., 2002; Parent et al., 2002; Jin et al., 2003; Sundholm-Peters et al., 2005; Ohab et al., 2006; Yamashita et al., 2006; Cayre et al., 2009; Young et al., 2011). It appears that the cells in general no longer migrate in a chain formation and carry on individually, with some reports describing an increase in progenitor numbers without an effect on numbers of cells in the RMS (Zhang et al., 2001b), while others report that the response to injury is at the expense of the RMS population (Jin et al., 2003; Goings et al., 2004). This change in migration is the direct result of chemoattractive cues expressed from the injury site. As detailed further below, chemokines and their receptors can attract neuroblasts from the RMS. For example, Stromal cell-derived factor-1/CXCL12 and its receptor CXCR4 are upregulated at the injury site (Imitola et al., 2004; Robin et al., 2006) and expression of several other chemokines and their receptors are upregulated on adult NSPCs by inflammatory cytokines, such as IFNγ and TNFα (Turbic et al., 2011).

In general ischemia models have demonstrated production of new neurons from the SVZ in damaged cortex or striatum, while injury of the cortex usually promotes the generation of astrocytes and microglia/macrophages at the site of injury, with few or no new neurons produced (Ramaswamy et al., 2005; Richardson et al., 2007; Kreuzberg et al., 2010; Blizzard et al., 2011; Zhang et al., 2011). Neurodegenerative disease models, such as Parkinson's disease (PD) models, have also demonstrated migration of SVZ NPCs to the site of damage, with production of neurons in some cases but not others (Cooper and Isacson, 2004; Kadota et al., 2009; Jing et al., 2012). The production of astrocytes and oligodendrocytes near the injury site may be a result of expression of repressors of neuronal fate (Kernie et al., 2001; Shear et al., 2004; Buffo et al., 2005). Further, as detailed below, administration of a variety of factors promotes neurogenesis and/or gliogenesis in these normally non-neurogenic sites. The SVZ-derived NSPC response and the new cells they produce is quite variable, depending on injury type, region of neural damage, species and likely a range of other factors that remain to be defined.

There is little/no evidence that SGZ hippocampal NSPCs can migrate away from their normal GCL fate; however they do respond to neural damage (Dash et al., 2001; Chirumamilla et al., 2002), even if the hippocampus is not directly affected by damage, such as in cortical impact models (Kernie et al., 2001). This leads to alterations in neurogenesis that can produce altered cognition and/or effects on memory and anxiety. Little is currently known about how this neurogenesis is altered, but will be touched on below.

## Transcription factor regulation of NSPC function

Factors intrinsic to the cell participate in multiple roles of neurogenesis from proliferation to differentiation. Generally, transcription factors are the most widely studied intrinsic factors researched in adult NSPCs and many are similar to programs for neurogenesis during development. However, while a substantial amount of information is known about the role that different transcription factors play in modulation of NSPC biology, much less is known about extrinsic factors that can be used to alter expression of the transcription factors to produce desired specific functional outcomes. This section will discuss the expression and roles of some transcription factors that may be useful therapeutic targets. Later sections will discuss effects of different extrinsic growth factors and cytokines, with particular reference to their role following neural damage, although there are currently few links described between these factors and expression of specific transcription factors in the adult NSPC or newborn neural cells.

### SVZ

Under basal conditions, NSPCs produced in the SVZ go on to form neuroblasts that migrate from the lateral ventricles along the rostral migratory stream (RMS) to the OB where they differentiate into neurons of the OB. The formation and proliferation of NSPCs is dependent on the Sox family of genes, in particular *Sox2*. The zinc-finger protein Ars2 (arsenite-resistant protein 2) controls the multipotent progenitor state of NSCs through activation of *Sox2* (Andreu-Agullo et al., 2012). c-Myb is required for maintenance of the neural stem cell niche, promoting expression of *Sox2* and *Pax6* and subsequent proliferation (Malaterre et al., 2008). Epigenetic pathways can also control proliferation; recently phosophorylation of the histone H2AX which was shown to limit proliferation and overall neurogenesis (Fernando et al., 2011). New neurons migrating from the RMS to the OB primarily become GABAergic granule neurons that provide lateral inhibition between mitral and tufted cells. A minority of the new neurons become periglomerular neurons that are involved in lateral inhibition between glomeruli, and a small number of these cells are dopaminergic. This differentiation is under transcriptional control and proneural basic-helix-loop-helix (bHLH) transcription factors control neuronal fate commitment in NSPCs. For example, type C cells of the SVZ fated to become GABAergic interneurons in the OB express *Ascl1* (Kim et al., 2007). *Ngn2* and *Tbr2* are expressed in dorsal SVZ progenitors that become glutamatergic juxtaglomerular neurons (Brill et al., 2009), while *Sp8* is required for parvalbumin-expressing interneurons in the OB (Li et al., 2011). Dopaminergic periglomerular cells in the OB express *Pax6* and *Dlx-2* (Doetsch et al., 2002; Hack et al., 2005; Brill et al., 2008).

### SGZ

Hippocampal neurogenesis involves radial and horizontal NPCs first transitioning to intermediate progenitors and then on to immature dentate granule neurons. When the new neurons mature they make large mossy fiber projections with CA3 pyramidal neurons (Freund and Buzsaki, 1996). *Sox2* has a role in maintaining the precursor pool via Sonic hedgehog (Shh) in adult SGZ and a role in proliferation of NPCs along with *Pax6* and CCAAT/enhancer binding protein β (C/EBPβ) (Maekawa et al., 2005; Favaro et al., 2009; Ehm et al., 2010). Transcription factors have a large role in the differentiation of SGZ NSPCs. *Neurog2* and *Tbr2* are expressed in NSCs destined to become glutamatergic neurons in the hippocampus (Kim et al., 2007, 2011a; Ozen et al., 2007), while over-expression of *Ascl1* produces oligodendrocytes (Jessberger et al., 2008).

Intrinsic factors are also necessary for the maturation and survival of newly born neurons. For example, *Prox1* (Lavado et al., 2010), *NeuroD* (Gao et al., 2009b; Kuwabara et al., 2009), and Kruppel-like factor 9 (Scobie et al., 2009) play important roles in survival, while cyclic response element binding protein (CREB) signaling is required for maturation and integration into the network. Interestingly, CREB activates miR-132 which regulates dendrite maturation in newborn dentate gyrus granular neurons (Magill et al., 2010). There is limited knowledge of the changes in SGZ intrinsic factors following injury; however neurogenesis is increased in the SGZ following injury. Following ischemia this increase can result from an upregulation of phosphorylated CREB (Boneva and Yamashima, 2012). In a primate model of ischemia, pro-neuronal transcription factors are expressed including *Emx2, Pax6*, and *Ngn2* (Tonchev and Yamashima, 2006). Intriguingly, in models of neurodegeneration SGZ proliferation is decreased; as shown in a rat model of Huntington's disease, SGZ progenitor cell proliferation is decreased due to an increase in *Sox2*-positive quiescent stem cells and a decrease in CREB signaling (Kandasamy et al., 2010).

Thus, many transcription factors have been shown to play a role in NSPC function, though few have been directly targeted by infusion of exogenous factors as a means to promote specific *in vivo* NSPC function and fate. Will this even be possible *in vivo* without genetic modification, such as by use of viral expression vectors? In many cases, particular exogenous factors were chosen for *in vivo* examination based on their known effects on induction of desired transcription pathways in other systems. However, in most instances, only single factors have been infused. This is in contrast to work being performed in the embryonic stem (ES) cell and induced pluripotent stem (iPS) cell field, in which specific transcription factors are induced by addition of extrinsic factors for defined periods of time and in a specific sequence to allow production of desired neural cell types. For example, such a system has been used to generate floor plate cells which can subsequently generate mesencephalic dopaminergic neurons (Denham et al., 2012). Of course, such specific transcriptional regulation by exogenous factors is possible in the highly defined ES/iPS tissue culture environment but much more difficult *in vivo*, where there are many competing endogenous factors regulating NSPC function under normal conditions and even more so following neural injury. Further, the question of which transcription factors would be worth targeting for potential therapeutic effects is still open. Promoting a glial vs. a neuronal fate is relatively easy; for example infusion of BMP4 induces SMAD1/5/8 phosphorylation and subsequent astroglial fate (Cate et al., 2010). Induction of specific neuronal fates has largely not been addressed. SVZ NSPCs predominantly generate OB interneurons under normal conditions; however following neural injury the SVZ NSPCs can alter their normal fate, even in the absence of exogenous factor delivery, to become glial cells or adopt the specific neuronal type lost due to damage, such as striatal medium spiny neurons (Parent et al., 2002). Therefore, factors in the local damaged environment may be sufficient to direct final neuronal fate under some conditions, suggesting that adult SVZ NSPCs are not necessarily fate restricted and that defining factors to induce specific transcription factors, such as bHLH proneural genes, may be beneficial.

## Extrinsic factors that regulate NSPC function following neural damage

While many extracellular factors, ranging from growth factors to morphogens to extracellular matrix to cell:cell/receptor-mediated interactions have been shown to play a role in adult NSPC biology *in vitro* and/or *in vivo*, some have been more well characterized than others. This section will cover the predominant factors, as well as some less well characterized factors, shown to regulate normal adult NSPC biology and those shown to play a role following neural damage.

### Growth factors

Following ischemia and traumatic brain injury, expression of various growth factors such as epidermal growth factor (EGF), brain-derived neurotrophic factor (BDNF), nerve growth factor (NGF), fibroblast growth factor 2 (FGF2), glial cell line-derived neurotrophic factor (GDNF), insulin-like growth factor 1 (IGF-1), pituitary adenylate cyclase-activating polypeptide (PACAP), and vascular endothelial growth factor (VEGF) are increased and modulate neurogenesis and NSPC biology. Additional exogenous application further augments NSPC proliferation and sometimes survival (Watanabe et al., 2004; Baldauf and Reymann, 2005; Tureyen et al., 2005; Dempsey and Kalluri, 2007; Schabitz et al., 2007; Johanson et al., 2011; Lu et al., 2011). This section will focus on factors that have been shown to regulate NSPC responses *in vivo* and commence with an examination of two of the most potent mitogens for increasing numbers of NSPCs, EGF, and FGF2.

#### EGF

EGF is a NSPC mitogen that is commonly used to promote neurosphere growth and maintenance in cell culture (Reynolds and Weiss, 1992). Neurospheres are primarily derived from the transit-amplifying (Type C) progenitor cells in the SVZ that normally produce the neuroblasts that migrate to the OB. These cells express the EGF receptor (EGFR) and when exposed to exogenous EGF can be induced to a multi-potent state capable of generating neuronal and glial cells (Doetsch et al., 2002).

Local production of EGF in the SVZ appears to be required for normal maintenance of the proliferative NSC pool in the SVZ, and this expression is maintained by dopaminergic innervation of striatal neurons (O'Keeffe et al., 2009; O'Keeffe and Barker, 2011). Activation of dopamine receptors using the dopamine receptor agonist pramipexole (PPX) augmented neurogenesis in a PD model by upregulating expression of the EGFR (Winner et al., 2009). Furthermore, the injury-induced proliferative response of SVZ NSPCs at least partly involves upregulation of the EGFR on these cells, thus enhancing their responsiveness to EGF (Alagappan et al., 2009). However, transforming growth factor alpha (TGFα), which also binds to the EGFR, is expressed at higher levels in the adult brain than EGF (Seroogy et al., 1993) and so TGFα may be the normal ligand for regulating NSPC function *in vivo*.

Given the mitogenic properties of EGF for NSPCs, infusion of EGF into the lateral ventricles following neural damage has been used to increase proliferation of NSPCs and attempt to increase their contribution to neural repair. This has led to mixed results, depending upon the injury/disease model used and possibly the species in which it was examined. EGF infusion for 6 days into the normal adult mouse forebrain increased the number of NSPCs at the SVZ, promoted their migration into the parenchyma (cortex, striatum, and septum), and resulted in 25% surviving for at least 7 weeks; however the majority of cells that differentiated were astrocytes with a small percentage of neurons and oligodendrocytes (Craig et al., 1996). Indeed, generally, EGF tends to promote an astroglial rather than neuronal fate, at least in rats (Kuhn et al., 1997). However, in mice, infusion of EGF into the lateral ventricles of the uninjured brain similarly increased SVZ NSPC proliferation and induced their migration into surrounding parenchyma, but most of the cells adopted an oligodendroglial fate, with a smaller percentage astroglial. This was further enhanced following a demyelinating lesion of the corpus callosum (Gonzalez-Perez et al., 2009). These cells may be a sub-population of SVZ NSPCs, the NG2+ oligodendrocyte progenitors, which express EGFR and are induced to migrate in response to EGF (Aguirre et al., 2005, 2007).

Conversely, in the damaged brain, such as following ischemia, EGF promoted SVZ NPC proliferation with subsequent production of neuroblasts in SVZ and striatum (Ninomiya et al., 2006), also it induced migration of doublecortin positive precursors and their subsequent long term (13 weeks) survival as parvalbumin-expressing interneurons (Teramoto et al., 2003). A similar infusion following traumatic brain injury also induced SVZ proliferation, as well as SGZ proliferation, at early timepoints but did not promote longer term (4 weeks survival) of the newborn SGZ cells, many of which had differentiated into astrocytes rather than neurons (Sun et al., 2010). Similarly, in a rat model of ischemia EGF alone induced proliferation and some NSPC migration but did not promote regeneration unless combined with a later administration of erythropoietin (Epo) (Kolb et al., 2007), with similar results obtained for infusion of TGFα in a PD model (Cooper and Isacson, 2004). Part of the discrepancy between differentiation outcomes may lie in rat:mouse species differences. In general, mouse studies have indicated that EGF infusion can promote proliferation, migration and at least some neuronal differentiation while rat studies have shown proliferation, some migration and differentiation into glial cells, if they are found to differentiate at all. This may be due to EGF-induced dysplasia in rat but not mouse SVZ (Lindberg et al., 2012). In rats, the EGF-induced NSPC proliferative response generates SVZ/lateral ventricle polyps, which contain a higher percentage of cells expressing the transcription factors *Sox2, Olig2*, markers of proliferative NPCs, as well as Id1, an inhibitor of neuronal differentiation, concomitant with decreased expression of the neuroblast marker doublecortin. This suggests that in rat, EGF promotes proliferation of more glial fated NPCs that do not migrate to the same extent as mouse NPCs exposed to EGF. However, combination of EGF plus other growth factors appears to overcome some of the limitations of infusion of EGF alone to some extent.

#### EGF plus FGF2

FGF2 is another potent NSPC mitogen, commonly used to induce proliferation of these cells in culture, usually in combination with EGF. Infusion of FGF2 by itself into the lateral ventricles of normal adult rats promotes NSPC proliferation and enhances neurogenesis in the OB (Kuhn et al., 1997). Following traumatic brain injury it has been shown to enhance SVZ and SGZ NSPC proliferation and increase the 4 weeks survival of newborn neurons (Sun et al., 2009), while FGF2 knockout mice show decreased hippocampal neurogenesis following seizure or ischemia (Yoshimura et al., 2001). In addition, conditional deletion of FGFR1, the major receptor for FGF2, decreases hippocampal neurogenesis and impairs memory consolidation (Zhao et al., 2007). Quite a few studies have used a combination of EGF and FGF2 infusion, which has generally promoted neural repair. In a rat global ischemia model, which led to loss of CA1 hippocampal neurons, EGF+FGF2 promoted proliferation of SVZ-derived cells which repopulated the damaged CA1 hippocampal neurons and promoted functional recovery (Nakatomi et al., 2002). Similar studies reported increased numbers of neural progenitors in hippocampus, as well as SVZ and hypothalamus (Oya et al., 2008). Comparable results were obtained following transient middle cerebral artery occlusion in rat, including increased neuronal numbers and survival (Tureyen et al., 2005). However, in an endothelin model of transient ischemia, while co-infusion of EGF+FGF2 promoted increases in SVZ neurogenesis, it decreased hippocampal neurogenesis and increased infarct volumes (Baldauf and Reymann, 2005) but the reasons for the different outcomes in these models is not clear. Increased proliferation and migration to dopamine deficient areas have also been observed following EGF+FGF2 infusion in a PD model (Winner et al., 2008).

#### Other factors that can promote NSPC proliferation

Although EGF and FGF2 are possibly the most potent NSPC mitogens, particularly for SVZ-derived cells, other factors can also promote increases in NSPC numbers, although most have yet to be tested in brain injury/disease models. Infusion of ATP into the lateral ventricles of mice increased numbers of transit amplifying progenitor cells (Suyama et al., 2012). A mechanism by which this occurs may involve induction of FGF2 and TGF synthesis (Jia et al., 2011). VEGF can augment SVZ progenitor proliferation, as well as migration (Wittko et al., 2009; Calvo et al., 2011). Other factors have been shown to enhance proliferation induced by EGF and/or FGF2 without having a notable effect by themselves, such as growth hormone (GH) (McLenachan et al., 2009).

IGFs also play a role in several NSPC functions. IGF1 has different effects in the SVZ and dentate gyrus SGZ. Peripheral administration promotes hippocampal dentate gyrus NSPC proliferation and increases subsequent dentate gyrus neurogenesis (Aberg et al., 2000) and survival (Cheng et al., 2001). GH receptor knockout mice, which are serum-IGF1-deficient, do not show any alterations in hippocampal neurogenesis (Ransome and Turnley, 2008b), which may indicate that local hippocampal/dentate gyrus production of IGF-1 is important in supporting ongoing hippocampal neurogenesis (Sun et al., 2005a). However, peripheral administration of GH promoted hippocampal but not SVZ NSPC proliferation (Aberg et al., 2009). Indeed, in the SVZ IGF1 does not seem to regulate proliferation, rather being more important for NSPC migration; IGF1 null mice show an abundance of neuroblasts in the SVZ that have failed to migrate to the OB (Hurtado-Chong et al., 2009). IGF2 regulates proliferation in the dentate gyrus in an Akt-dependent manner (Bracko et al., 2012) and promotes survival of newborn hippocampal neurons as well as regulating hippocampal dependent fear extinction (Agis-Balboa et al., 2011).

### Neurotrophins

Neurotrophin signaling mediated by BDNF interacting with TrkB receptors and to a much lesser extent NGF binding to TrkA, regulates several aspects of NSPC function. In the adult brain, TrkB expression and effects of BDNF are widespread but TrkA expression is very limited, primarily restricted to basal forebrain cholinergic neurons which project to the NGF-expressing hippocampus. Therefore, effects of BDNF in NSPC biology have been the most widely studied, although NGF infusion has been shown to promote the survival of normal newborn dentate granule cell neurons but not proliferation of their progenitors (Frielingsdorf et al., 2007). Intranasal delivery of NGF following focal ischemia in rats similarly did not promote proliferation of SVZ progenitors but enhanced subsequent newborn neuronal survival in the ipsilateral SVZ and injured striatum (Zhu et al., 2011).

*In vitro*, SVZ-derived neurospheres express TrkB and little/no TrkA or TrkC and addition of BDNF promoted a transient increase in newborn neuron numbers due to enhanced differentiation and neurite outgrowth, rather than a proliferative effect on the NSPCs (Ahmed et al., 1995). Further, BDNF is required for cultured hippocampal progenitor cells to adopt a neuronal fate (Bull and Bartlett, 2005).

In the dentate gyrus, TrkB is expressed predominantly by maturing but not proliferating neuroblasts (Donovan et al., 2008), which correlates with findings that BDNF is important for neuroblast migration, survival, and integration of new neurons. BDNF increases the number and survival of newborn neurons in the SVZ and OB (Kirschenbaum and Goldman, 1995; Zigova et al., 1998; Bath et al., 2008), in a p75 neurotrophin receptor-dependent manner (Young et al., 2007), and in striatum, caudate putamen and septum (Benraiss et al., 2001; Henry et al., 2007), dentate gyrus (Lee et al., 2002) as well as subcallosal neurons (Kim et al., 2011b). Knockdown of TrkB receptors and disruption of BDNF signaling resulted in decreased SGZ NSPC proliferation and neurogenesis, indicating that TrkB signaling may also have some proliferative effects in these cells (Li et al., 2008). Disruption of BDNF signaling also results in shorter dendrites and reduced spine formation, culminating in a lack of survival of newborn granule cells (Bergami et al., 2008; Gao et al., 2009a). In addition, conditional deletion of BDNF resulted in increased death of newborn neurons in mice following traumatic brain injury (Gao and Chen, 2009). However, conversely, enhanced long-term AAV-mediated BDNF expression in the hippocampus was shown to inhibit neurogenesis following ischemia in rats (Larsson et al., 2002). The reason for this discrepancy is unclear and further work is required to delineate the mechanisms by which BDNF regulates adult neurogenesis following neural damage. Given that neural activity promotes BDNF-mediated TrkB activation (Aloyz et al., 1999) and TrkB signaling is required for activity-dependent differentiation of hippocampal NSPCs, BDNF is likely to be a critical factor to promote NSPC-derived newborn neuron survival and integration into neural circuitry. However, if too much BDNF-stimulation is provided, this may be at the expense of NSPC numbers in some situations but this needs further study for confirmation.

BDNF combined with a mitogen is more effective in enhancing NSPC function and may be a better therapeutic option. Neurogenic NSPCs can be cultured from SVZ of adult humans but requires a combination of FGF2 and BDNF for robust growth (Pincus et al., 1998). This combination also appears to be required for creating a neurogenic environment in normally non-neurogenic regions *in vivo* (Chen et al., 2007a). In addition, co-infusion of BDNF plus EGF was more effective than either factor alone at promoting long term striatal neuron survival following ischemic injury in mice (Im et al., 2010).

### Morphogens

The morphogens Wnt, Shh, and Bone Morphogenic Proteins (BMPs) play a major role in neural development with some data to suggest they may also play a role in the NSPC response to neural damage.

#### Wnt signaling

While Wnts are endogenously expressed in adult neurogenic niches (Wexler et al., 2009), their expression is not upregulated following neuronal damage, such as stroke (Morris et al., 2007). Wnt signaling via β-catenin, promotes NSC proliferation to regulate their maintenance (Lie et al., 2005; Adachi et al., 2007). This appears to be by regulating symmetric vs. asymmetric division, promoting symmetric division during neural regeneration (Piccin and Morshead, 2011). Wnt signaling is negatively regulated by glycogen synthase kinase 3 beta (GSK3β) and inhibitors of GSK3β have been proposed as candidate targets for neural repair (Mao et al., 2009). Inhibition of β-catenin signaling by the amyloid beta peptide resulted in reduced neurogenesis from NPCs grown from Alzheimer disease brains, which also have increased levels of GSK3β (He and Shen, 2009). Further, downregulation of β-catenin in a rat stroke model inhibited striatal neurogenesis (Lei et al., 2008), while overexpression of Wnt3a following focal ischemia promoted NSPC proliferation, neurogenesis and functional recovery (Shruster et al., 2012). However, following traumatic brain injury in mice, β-catenin activity was upregulated in cortical NG2+ oligodendrocyte/astrocyte progenitor cells, suggesting that it also plays a role in gliogenesis following injury (White et al., 2010).

#### Shh

Intrathecal administration of Shh to rats following ischemia promoted SVZ NSPC proliferation and improved behavioral recovery (Bambakidis et al., 2012), while following spinal cord injury it promoted proliferation of nestin+ NSPCs surrounding the central canal leading to increased numbers of oligodendrocyte precursors and neurons (Bambakidis et al., 2003). Shh expression is upregulated in the SVZ and hippocampus following ischemia, potentially playing a role in increased proliferation in a Notch dependent manner (Sims et al., 2009; Wang et al., 2009); however in the hippocampus its expression was also upregulated in mature CA3 neurons, suggesting effects in mature neurons as well as progenitors (Sims et al., 2009). The source of Shh in the adult brain appears to be astrocytes. In the normal brain, astrocytes in neurogenic regions produce Shh and when transplanted they induce neurogenesis in non-neurogenic regions of the brain, such as cortex (Jiao and Chen, 2008). Following brain injury activated astrocytes upregulate Shh expression in response to pro-inflammatory stimuli, which subsequently promotes increased numbers of Olig2+ NSPCs (Amankulor et al., 2009); this may be via activation of quiescent endogenous cortical NSPCs derived from astrocytes (Ahmed et al., 2012). However, in different neuroinflammatory conditions, such as experimental allergic encephalomyelitis (EAE) or multiple sclerosis, the inflammatory response induces Shh in astrocytes as above but inhibits Shh-induced NSPC differentiation by subsequent downregulation of the Gli1 transcription factor (Wang et al., 2008).

#### Bone morphogenic proteins (BMPs)

The role of BMP signaling in NSPCs and their role following CNS injury has recently been reviewed (Sabo et al., 2009) and so will only be covered briefly here. In general, BMP signaling inhibits neuronal and oligodendroglial differentiation of NPCs and promotes astrogliogenesis, during development (Gross et al., 1996) and in the adult (Lim et al., 2000), with somewhat different effects at different stages of development (Mehler et al., 2000). However, expression of the BMP inhibitor noggin in the SVZ (Lim et al., 2000) or SGZ (Bonaguidi et al., 2008) can obstruct this cascade and promote neurogenesis. Further, BMP signaling promotes non-neurogenic parenchymal astroglial cell fate while leukemia inhibitory factor (LIF), which also promotes astrogliogenesis, promotes cells of an astroglial progenitor cell phenotype (Bonaguidi et al., 2005). Demyelination induced expression of the BMP antagonist chordin in the SVZ, induced glial fate in neuroblasts to generate new oligodendrocytes in the corpus callosum (Jablonska et al., 2010). Conversely, BMP signaling in SVZ NSCs but not NPCs is required to promote a neurogenic rather than oligodendrogliogenic cell fate (Colak et al., 2008).

BMP signaling inhibits NPC proliferation (Gajera et al., 2010) and while inhibition of the BMP pathway increases neurogenesis initially, it eventually results in depletion of the NSC pool, leading to decreased neurogenesis in the dentate gyrus (Mira et al., 2010). This indicates that a fine balance of BMP signaling vs. inhibition is required to regulate appropriate NSC numbers and subsequent cell fate.

Given that BMP signaling promotes an astroglial fate and inhibits oligodendrogliogenesis and neurogenesis, the effect of inhibition of BMP signaling by infusion of noggin into the lateral ventricles has been examined in various models of neural injury. In cuprizone-induced demyelination models noggin infusion inhibited the cuprizone induced upregulation of BMP4 and its signaling pathways, decreased SVZ astrocyte numbers, increased oligodendrocyte numbers and promoted remyelination of the corpus callosum (Cate et al., 2010; Sabo et al., 2011), possibly by regulation of Olig2 function (Chen et al., 2012). Noggin infusion also produced similar results in a model of intraventricular hemorrhage (Dummula et al., 2011). In a brain injury model, noggin was found to be produced by reactive astrocytes and similarly promoted oligodendrocyte fate (Hampton et al., 2007). It was also recently shown that Bcl2 regulates neurogenesis in a striatal injury model, by increasing β-catenin expression and decreasing BMP4 expression, in a noggin independent fashion (Lei et al., 2012).

### Erythropoietin (Epo)

Epo is a cytokine better known for its regulation of erythrocyte production. However it also has a number of functions within the CNS and on NSPC biology. Epo is expressed in the developing and adult SVZ (Shingo et al., 2001) and is required for endogenous embryonic and adult SVZ and SGZ neurogenesis (Tsai et al., 2006; Chen et al., 2007b). It promotes neurogenesis of SVZ NSPCs at the expense of multipotent progenitors in the normal rodent brain (Shingo et al., 2001) but in the SGZ this effect is transient, briefly increasing neuronal progenitor numbers but with no long term enhancement of survival (Ransome and Turnley, 2007). One of the mechanisms by which it may do this is by upregulation of Suppressor of Cytokine Signaling-2 (SOCS2) in NSPCs (Wang et al., 2004b). SOCS2, an intracellular regulator of cytokine signaling, promotes embryonic SVZ neurogenesis (Turnley et al., 2002; Scott et al., 2006), while in the hippocampus it promotes NSPC-derived newborn neuron survival (Ransome and Turnley, 2008b). It also promotes axonal growth of hippocampal neurons (Ransome and Turnley, 2008a), which may be a factor contributing to its enhanced neuroprotective effects following neural injury.

Following neural damage Epo has been found to be both neuroprotective and to promote neurogenesis (Wang et al., 2004a). Epo expression is induced/upregulated by hypoxia and thus many studies have examined its role post-ischemia or in ischemia/hypoxia models, although it has also been shown to be effective following traumatic brain injury. Conditional knockdown of the Epo receptor reduced post-stroke neurogenesis, with reduced proliferation and stroke-induced neuroblast migration to the cortex (Tsai et al., 2006). The Epo-induced NSPC migration appears to be indirect, rather inducing expression of matrix metalloproteases in endothelial cells, which in turn induced NSPC migration (Wang et al., 2006). Peripheral administration of Epo promotes NSPC proliferation, neurogenesis, oligodendrogenesis, and neurovascular remodeling following traumatic brain injury in rats, enhancing functional outcome (Lu et al., 2005; Zhang et al., 2010; Meng et al., 2011; Ning et al., 2011; Xiong et al., 2011). Inhibition of proliferation by infusion of AraC into the ventricles inhibited Epo-induced dentate gyrus neurogenesis and recovery of spatial learning (Zhang et al., 2012). Epo has also been shown to be neuroprotective in disease models, such as PD, in which it promoted SVZ NSPC proliferation and migration to the damaged striatum (Kadota et al., 2009).

One of the problems associated with use of Epo as a therapeutic for promotion of neural regeneration is that it generally increases the hematocrit at doses required for promotion of neurogenesis (e.g., 5000 U/kg). While this has not presented major issues for use in animal models, it raises concerns for potential clinical use. Therefore, several Epo derivatives have been developed to try to promote neuroprotective effects while avoiding effects on erythrocyte production. Carbamylated Epo does not bind to classical Epo receptor and does not stimulate erythropoiesis but does promote SVZ and dentate gyrus proliferation and neuronal differentiation of adult NSPCs (Wang et al., 2007; Leconte et al., 2011), while a different non-erythropoietic derivative asialo-Epo promoted SVZ-derived oligodendrogenesis (Kako et al., 2012). A peptide agonist of Epo, Epobis, has also recently been developed and shown to promote neuron survival and neurite outgrowth, but its effects on neurogenesis have yet to be determined (Pankratova et al., 2012).

### Chemokines and cytokines

Chemokines form a family of small (8–14 kD), mainly basic, secreted molecules that are primarily known for regulating chemoattraction of immune cells to sites of tissue damage. They have been reported to have widespread non-immunological effects in the CNS, including regulation of neural cell proliferation, migration, survival, and synaptic transmission and can act in a paracrine or autocrine manner (Bajetto et al., 2002; Cartier et al., 2005).

Pro-inflammatory cytokines, such as interferon gamma (IFNγ) and tumor necrosis factor alpha (TNFα) appear to be major regulators of chemokine and chemokine receptor expression in many tissues (Hiroi and Ohmori, 2003; Suyama et al., 2005). In the adult brain there is basal expression of chemokines, especially in neurogenic regions, while treatment with IFNγ and TNFα can significantly increase the expression of specific chemokines including CXCL1, CXCL9, and CCL2 (Turbic et al., 2011).

Adult NSPCs express a range of chemokine receptors and chemokines are expressed in different brain regions, with the highest levels in the OB, suggesting an as yet largely unexplored role for chemokines in regulating basal adult NPC migration (Turbic et al., 2011). Neurospheres derived from adult mouse SVZ-derived NPCs have been shown to express a range of chemokine receptors, including CCR1-8, 10 and CXCR1-6 (Tran et al., 2004). Functionally, specific chemokines, such as CXCL12/CXCR4 can promote NPC migration (Imitola et al., 2004; Tran et al., 2004; Dziembowska et al., 2005) and proliferation (Tran et al., 2004) or survival (Krathwohl and Kaiser, 2004; Dziembowska et al., 2005) *in vitro*. Following neural damage, NPC migration to site of injury is promoted by CXCL12 (Imitola et al., 2004; Itoh et al., 2009) and is mediated at least in part, by induction of metalloprotease expression in the NPCs (Barkho et al., 2008). CCL2, CCL3, and CXCL1 also promote NPC migration to striatum following quinolinic acid lesion (Gordon et al., 2009).

The gp130-associated cytokines, ciliary neurotrophic factor (CNTF) and leukemia inhibitory factor (LIF), activate Janus kinase [JAK/signal transducer of transcription 3 (STAT3)], mitogen activated protein (MAP) kinase and PI-3K/Akt pathways following ligand binding. These cytokines have been shown to regulate NSC proliferation and differentiation (Turnley and Bartlett, 2000; Heinrich et al., 2003; Kamimura et al., 2003; Ernst and Jenkins, 2004). IFNγ, which signals via STAT1, and IFNβ which does not, both inhibit cultured adult NPC proliferation, but only IFNγ promotes neuronal differentiation (Wong et al., 2004; Lum et al., 2009). Specifically in the dentate gyrus, the activation of STAT3 from CNTF appears to be essential for the formation and maintenance of the NSCs (Muller et al., 2009).

## Other factors that play a role in NSPC function that may be targeted for neural repair

There are factors involved in neurogenesis other than extrinsic growth factors and cytokines, as discussed above. Some of these include membrane bound molecules, neurotransmitters and their receptors and blood vasculature.

### Eph/ephrin signaling and NSPCs—a new target to promote NSPC proliferation, survival, and migration

The Eph family of receptor tyrosine kinases and their ligands, the ephrins, are membrane bound molecules that signal bi-directionally, are generally involved in cell or axon guidance by repulsive mechanisms and which play a role following neural injury (reviewed in Goldshmit et al., 2006b). They have been receiving increasing interest of late as they have been shown to play major roles in inhibition of regeneration in the CNS following neural injury or disease, with deletion or blocking of various Ephs or ephrins promoting neural repair (Goldshmit et al., 2004, 2006b, 2011; Rodger et al., 2004; Liu et al., 2006; Fabes et al., 2007; Overman et al., 2012; Van Hoecke et al., 2012). EphA4 also plays a role in regulation of inflammation following neural injury (Munro et al., 2012), which could have secondary effects on NSPC responses.

Eph receptor signaling also regulates several aspects of NSPC biology but as yet has not been targeted to enhance NSPC responses following injury. The EphB2/ephrin-B2 pathway enables formation of the chain migration from the SVZ to the OB (Conover et al., 2000) and also controls the conversion of ependymal cells to astrocytic NSPCs (Nomura et al., 2010). EphB2 signaling via ephrin-B1 is also required for development of the dorsal half of the dentate gyrus, where it controls migration of the dentate progenitor cells (Catchpole and Henkemeyer, 2011). Ephrin-A2 signaling via EphA7 inhibits SVZ NSPC proliferation and subsequent neurogenesis (Holmberg et al., 2005), while conversely ephrin-A5 signaling via EphA7 induces their apoptosis (Depaepe et al., 2005). EphB2 and ephrin-B signaling however promotes SVZ NSPC proliferation, decreases migration, and promotes neuronal differentiation (Katakowski et al., 2005), with ephrin-B1 expression in SVZ NSPCs critical for maintenance of the proliferative NSPC state (Qiu et al., 2008), a role also played by EphA4 (Khodosevich et al., 2011). In addition, EphB3 is expressed by adult SVZ NSPCs and neuroblasts and EphB3 and ephrin-B3 knockout mice show increased neonatal and adult SVZ NSPC proliferation, indicating that this pathway normally inhibits their proliferation (Theus et al., 2010; Del Valle et al., 2011; Doeppner et al., 2011). Infusion of soluble ephrin-B3 into the lateral ventricles reversed the proliferation defect in ephrin-B3 knockout mice but not the EphB3 receptor knockout mice (Theus et al., 2010).

In the hippocampus, EphB1 and ephrin-B3 promote hippocampal NSPC proliferation and migration, as well as cell polarity (Chumley et al., 2007), while ephrin-A5 promotes hippocampal NSPC proliferation and neurogenesis, at least partly through regulation of the normal vascular system (Hara et al., 2010), which requires normal EphA4 (Goldshmit et al., 2006a) and EphB4 signaling (Colin-Castelan et al., 2011). Ephrin-B2, expressed by hippocampal astrocytes in the SGZ, induces neuronal differentiation of EphB4-expressing hippocampal NSPCs and this effect involves activation of β-catenin independent of Wnt signaling, with subsequent upregulation of proneural transcription factors (Ashton et al., 2012).

To date, little has been examined regarding the role of Eph/ephrin signaling following neural damage. Following traumatic brain injury, EphB3 expression was transiently downregulated, which may lift its inhibition of NSPC proliferation and this may be one of the mechanisms by which NSPCs are able to respond to injury, by increasing proliferation and survival (Theus et al., 2010). However, in ephrin-B3 knockout mice, while there was an increase in NSPC proliferation following ischemic injury, there was also enhanced cortical damage with an increased infarct volume (Doeppner et al., 2011) due to NSPC-independent effects of ephrin-B3, which will need to be taken into account when considering therapeutic use of Eph/ephrins. Conversely, in a model of PD infusion of soluble ephrin-A1 into the lateral ventricles, to activate EphA receptor/s, promoted SVZ NSPC proliferation and migration of these cells to striatum, where they subsequently differentiated in dopaminergic neurons (Jing et al., 2012).

### Other factors

Following injury, one of the early mediators of the injury response is upregulation of nitric oxide (NO). NO has been reported to have proliferative (Zhang et al., 2001a; Reif et al., 2004) or anti-proliferative (Packer et al., 2003; Park et al., 2003; Moreno-Lopez et al., 2004) effects on basal SVZ and SGZ NSPCs, as well as following injury (Zhang et al., 2001a; Zhu et al., 2003; Sun et al., 2005b). This apparent discrepancy in effect appears to lie largely in the source of NO production and the subsequent signaling pathways induced. If the NO derives from neuronal nitric oxide synthase (nNOS) then the effect is largely anti-proliferative (Packer et al., 2003; Moreno-Lopez et al., 2004; Sun et al., 2005b), but if derived from inducible NOS (iNOS) (Zhu et al., 2003; Carreira et al., 2010) or endothelial derived NOS (eNOS) (Reif et al., 2004) then the effects are largely proliferative. This likely relates to the location of NOS as well as the concentration of NO produced. Further, infusion of NO donors that induce MAP kinase signaling (Carreira et al., 2010) or cyclic guanosine monophosphate (cGMP) signaling, promotes proliferation and neurogenesis in normal and ischemic rats (Zhang et al., 2001a). Indeed, both pathways appear to be involved in NSPC proliferation in response to NO, with activation of the MAPK pathway at shorter timepoints (6 h) and activation of cGMP at later timepoints (24 h) (Carreira et al., 2012). Recent work has indicated that the NO-cGMP pathway is also an important mediator of the neuro-proliferative effects of other factors, such as Neuropeptide Y in the hippocampus (Howell et al., 2005; Agasse et al., 2008; Cheung et al., 2012) and also explains the increased proliferation and neurogenesis observed with infusion of phosphodiesterase inhibitors, such as sildenafil and tadalafil, which lead to increased cGMP levels and improve outcome in stroke models (Zhang et al., 2003, 2006). cGMP levels also appear to regulate NSPC fate during embryonic development, with high levels promoting neuronal differentiation and low levels non-neuronal fate (Gomez-Pinedo et al., 2010), however, whether this is the case in adult remains to be determined.

Endocannabinoid signaling has been shown to regulate migration and neurogenesis in both the SVZ and dentate gyrus (Aguado et al., 2005, 2007; Jiang et al., 2005; Goncalves et al., 2008; Gao et al., 2010; Hill et al., 2010; Wolf et al., 2010; Oudin et al., 2011a,b). Other molecules involved in NPC migration include polysialated neural cell adhesion molecule (PSA-NCAM) (Cremer et al., 1994; Hu et al., 1996; Chazal et al., 2000), Slit-Robo (Wu et al., 1999), and integrins (Jacques et al., 1998; Murase and Horwitz, 2002). In basal conditions, reelin is a detachment signal for neuroblasts from the RMS at the OB (Hack et al., 2002), while overexpression of reelin in a demyelinating lesion led to an increase in ectopic migration of neuroblasts at the lesion site (Courtes et al., 2011). Many of these factors signal via the Rho kinase pathway, which is a downstream regulator of NPC migration (Leong et al., 2011) and targeting this pathway may circumvent the large number of external signals that converge on this pathway to allow more precise control of directed NPC migration and possibly neuronal differentiation and survival. In the brain parenchyma NSPCs interact with blood vessels in the neurovascular niche (Shen et al., 2008; Tavazoie et al., 2008), neuroblasts can migrate along blood vessels (Honda et al., 2007) and use vessels to migrate radially into the cortex (Le Magueresse et al., 2012). Following ischemia SVZ neuroblasts migrate to the infarct in close association with blood vessels (Ohab et al., 2006; Yamashita et al., 2006; Thored et al., 2007; Kojima et al., 2010).

NMDA receptors expressed in neuroblasts along the RMS are crucial to the integration of these neurons in existing OB circuitry (Platel et al., 2010), with glutamate released from astroglial cells in the glial tube that surrounds the migrating neuroblasts. NMDA receptor activation in newly born dentate gyrus granule cells also increases survival. Initial GABA depolarization promotes maturation of neurons in the dentate gyrus and OB (Saghatelyan et al., 2005; Ge et al., 2006) and this depolarization and subsequent Ca^2+^ influx are required for dendrite initiation and elongation (Gascon et al., 2006). Agrin signaling is also necessary for integration and survival of newborn neurons in the OB: a loss of agrin leads to improper synapse formation while overexpression of agrin results in an increase in dendritic spines (Burk et al., 2012).

The migration distance for new neurons from the SGZ is relatively short as they travel into the granular layer above the SGZ, where guidance molecules may control this movement. NMDA receptor signaling is required for the proper migration of newborn granular cells in the dentate gyrus (Namba et al., 2011). This is achieved through the activation of Disrupted-in-schizophrenia (DISC1), as neurons without DISC1 migrate further into granular layer and into the molecular layer (Duan et al., 2007; Namba et al., 2011). DISC1 also controls the dendritic maturation of newborn granule cells through GABA depolarization of NKCC1 and activation of the Akt-mTOR pathway (Duan et al., 2007; Kim et al., 2012), while Reelin regulates migration and dendritic development of adult-generated hippocampal neurons (Teixeira et al., 2012). Whether targeting any of the above factors will enhance NSPC responses following neural injury largely remains to be determined.

## Conclusions

Many factors regulate the biology of NSPCs at different stages along their growth and maturation cycle. To date, the majority of studies which have administered extrinsic growth factors or cytokines have concentrated on the effect of a single factor at a time. However, some studies are now emerging where combinatorial effects of different factors have been examined. It will not be sufficient to only increase the number of NSPCs produced by administration of a mitogen. While this can indeed increase neurogenesis, the number of surviving and functionally integrated neurons (or oligodendrocytes) is still very limited. It would be prudent to combine use of mitogens with other factors that enhance neurogenesis or oligogliogenesis respectively, as well as factors that enhance their subsequent survival and integration. Which factors are chosen to combine will likely depend on the injury or disease model to be examined, whether neurons and/or oligodendrocytes need to be replaced and may also be species dependent to a certain extent, although the combination of a mitogen such as EGF and a survival factor, such as BDNF may be generally beneficial. Some factors, such as Epo, have multiple actions, including NSPC proliferation, neuronal differentiation, and oligodendrocyte maturation, as well as being neuroprotective, which makes them particularly attractive as potential therapeutics for treatment of the damaged brain. Most of the work described above was conducted in rodents and to date little work has been performed following neural injury in brains of gyrencephalic species, such as non-human primates (Tonchev et al., 2003a,b, 2005, 2007). Sheep models are also being developed to allow such questions to be answered in a model more relevant to human brain damage (e.g., Wells et al., 2012). Human clinical trials are also underway to test therapeutic effectiveness of a range of extrinsic factors, such as Epo, following brain injury. For a complete list see www.clinicaltrials.gov. Two decades since their discovery in mammalian brains, facilitation of the NSPC response to brain damage by administration of extrinsic factors still holds great promise of therapeutic potential, although much ground still remains to be covered.

### Conflict of interest statement

The authors declare that the research was conducted in the absence of any commercial or financial relationships that could be construed as a potential conflict of interest.
